# MOLGENIS/OMX for multi-omics and personalized medicine

**DOI:** 10.1186/2043-9113-5-S1-S5

**Published:** 2015-05-22

**Authors:** Morris Swertz, K Joeri van der Velde

**Affiliations:** 1 Genomics Coordination Center, Department of Genetics, University Medical Center Groningen, 9700 RB Groningen, the Netherlands

## Characterisation

Tool, genomics, personalized medicine, meta-data, data, annotation, integration, visualization, open source/free.

## Description

MOLGENIS/omx [1] is a generic toolbox for multi-omics and personalized medicine studies such as EU-Panacea, EU-BioSHaRE, EU-BioMedBridges, NL-String of Pearls, and CTMM/Triumph. OMX comes with a simple Excel format to upload data; a powerful data explorer to find and filter data; a genome browser; an R statistical programming interface for bioinformaticians and integrated annotation tools for Chembl, Ensembl, OMIM, etc. Increasingly bigger datasets are required for epidemiological and genetic analysis and hence, it has ‘big data’ stores connected using VCF and Elastic searches. MOLGENIS/omx gave rise to a variety of web applications in the life science domain, including omics data warehouses, patient mutation registries, and clinical data integration platforms. For example, the WormQTL^HD^ database [2] contains over 60 omics data sets contributed by the international *C. elegans* research community. Patient registries such as those developed for MVID [3], Dystrophic EB, and CHARGE syndrome enable clinicians to enter data about patient phenotypes and causal mutations. MOLGENIS/omx most recent application is to facilitate interpretation of the massive amounts of genomics data, including eQTL pathogenicity predictions, and/or clinical interpretation of NGS data in research consortia and clinical labs.

## Status of development

Version 1.2.0.

## Users

10 known installations.

## Links

http://github.com/molgenis/molgenis, http://www.molgenis.org

**Figure 1:**
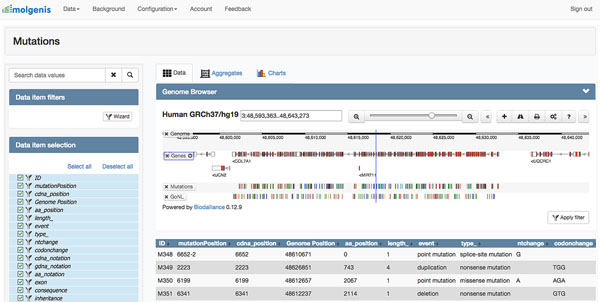
MOLGENIS/omx genome browser in the COL7A mutation database.
